# RF injection scanning tunneling spectroscopy of a superconducting NbSe_2_ surface

**DOI:** 10.1038/s41598-025-07203-2

**Published:** 2025-07-01

**Authors:** Md. Arafat Ali, Zhipeng Wang, Mohammad Ikram Hossain, Ferdous Ara, Syed Mohammad Fakruddin Shahed, Tadahiro Komeda

**Affiliations:** 1 Department of Chemistry, Graduate School of Science, Tohoku University, 6-3, Aramaki Aza-Aoba, Aoba-ku, Sendai, 980-8578 Japan; 2 Institute of Multidisciplinary Research for Advanced Materials (IMRAM, Tagen), Tohoku University, 2-1-1, Katahira, Aoba-Ku, Sendai, 980-0877 Japan; 3 Center for Spintronics Research Network, Tohoku University, 2-1-1 Katahira, Aoba-ku, Sendai, 980-8577 Japan

**Keywords:** Scanning tunnelijng microscopy (STM), Photo-assisted-tunneling (PAT), Radio frequency (RF) signal, NbSe_2_, Superconducor, Superconducting properties and materials, Two-dimensional materials, Scanning probe microscopy

## Abstract

NbSe_2_ is a transition metal dichalcogenide with a two-dimensional nature, showing superconducting (SC) and charge density properties. Moreover, a 1T phase can be made in the film, which has a different property from the bulk dominant 2 H phase, in which Mott insulator behavior is actively discussed. We observed the surface with STM at 400 mK and injected RF signal (1 GHz and 15 Hz) at the tunneling junction. We detected two quasi-particle (QP) states at the end of the SC gap, which split with the increase of the RF power specified by the electric field at the tunneling junction, V_AC_. A previous STM experiment with 65 GHz RF on a vanadium surface observed multiple replicas of the QP peak. However, our experimental result using 1 GHz RF shows a widening of QP features with two enhanced peaks shifted by ~ ± eV_AC_ from the original QP positions. The behavior was well reproduced by a simulation using a well-known Tien-Gordon model, whose results indicate that the disappearance of multiple peaks is due to the low frequency of the RF signal. In addition, two enhanced peaks at ~ ± eV_AC_ are deduced from the Bessel function behavior. The energy shift from the original peak linearly changes with eV_AC_. We apply this technique to examine the property change at the domain boundary of the 2 H and 1T phase of the NbSe_2_ surface. We found the superconducting gap decreases when we move the tip from the 2 H domain into the 1T domain. Moreover, the injection of RF splits a QP peak into two enhanced peaks, whose energy separation is linear with the electric field at the RF generator for both phases. However, the linear energy separation with V_AC_ shows different coefficients between the 2 H and 1T phases. We conclude that the different coefficient is due to the change of actual V_Ac_ on the two domains originating from a different dielectric constant and shielding efficiency for the electric field of RF.

**Supplementary Information:**

The online version contains supplementary material available at 10.1038/s41598-025-07203-2.

## Introduction

Two-dimensional materials attract attention to quantum information technology; especially topological superconductor states are explored for a search of Majorana state that is indispensable for a quantum computer realization^1^. NbSe_2_ is a transition metal dichalcogenide (TMD) showing superconductivity (SC)^2^. It also shows the charge density wave (CDW) state, and the coexistence of SC and CDW has been an intensive research target. Although the 2 H phase dominates in the bulk state, the 1 T phase is also observed in a film state. In the 1 T phase, NbSe_2_ exhibits intriguing properties due to strong electron correlations, including Mott insulating states^3^. However, recent calculations revealed that the correlation gap was due to charge transfer^4–6^, not due to a Mott-Hubbard gap, as previously assumed^7^. For the SC property, the bulk NbSe_2_ can be described as either a strongly anisotropic s-wave superconductor or a two-gap superconductor^8–13^, but the investigation into how the electronic state changes in the 1 T phase is still ongoing. One of the difficulties in the 1 T phase investigation is that it is not the majority phase in the bulk. Thus, developing a surface-sensitive technique to unveil these issues is also essential.

A combination of electromagnetic wave and the scanning probe microscopy has attracted increasing attention. The nanoscale properties of a tunneling region can be identified by employing light waves of different wavelengths. Radiofrequency (RF) signals employed in conventional electron spin resonance spectroscopy can be used with the scanning tunneling microscope (STM) to detect and manipulate single-electron spins, as recently demonstrated in experiments^14–20^. For the working principle of such a single spin detection, an oscillation of a spin by the AC electric field of the RF signal at the tunneling junction (we call V_AC_ hereafter) is attributed. In addition, the scanning tunneling spectroscopy (STS) features of a sharp peak width, like a surface state of coinage metal surfaces, and an inelastic tunneling component of spin excitation, show intriguing changes upon RF injection^21^. The STS can be used to estimate the amplitude of the electric field at the tunneling junction. Hereinafter, we refer to the effect of STS on the RF electric field as the V_AC_ effect.

The phenomenon resulting from the injection of an RF signal into a superconductor–insulator–superconductor (SIS) tunneling junction is known as photon-assisted tunneling (PAT) and has been extensively studied for a long time^22–24^. The pioneering work of Tien and Gordon revealed that the sharp tunneling feature of an SIS at the edge of a superconductor gap changes into a ladder-like feature owing to the inelastic tunneling process during which energy is gained (lost) by tunneling electrons through the absorption (emission) of multiple photons^24^. Recently, the PAT measurements have been combined with STM platforms, and phenomena such as multiple Andreev reflections have been observed in various physical systems^25,26^. However, the application of STM-PAT research for multiple systems has just started.

Herein, we investigate a NbSe_2_ surface containing the 2 H phase with 3 × 3 charge density wave (CDW) reconstruction together with pulse-created 1 T phase of √13 × √13 superstructure at the sample temperature of 400 mK and injected RF signal (1 GHz and 15 Hz) at the tunneling junction. We observe two quasi-particle (QP) states at the end of the SC gap, which split with the increased power of the RF. However, unlike a previous STM exexperiment for the vanadium surface where multiple replicas of the QP peak^26^, we observe a smooth widening of the QP peak with two enhanced peaks at the energy ~ eV_AC_ shifted from the original position. The behavior was well reproduced by a simulation using the PAT model, revealing the mechanism of the disappearance of the sharp peaks. In addition, we successfully showed the origin of the two enhanced peaks. We apply this technique to investigate property changes at the boundary of 2 H and 1 T phases in the NbSe_2_ surface. We found the superconducting gap decreases in energy when we move the tip from the 2 H domain into the 1 T domain. Moreover, the linear energy shift of the RF-induced peaks with RF power shows different coefficients between 2 H and 1T. We concluded that this change is deduced from a difference in the actual V_AC_ on the two domains, originating from different dielectric constants formed by the insulating nature of the 1 T layer, resulting in a different shielding efficiency for the electric field of RF.

## Results and discussions

**Fig. 1:**
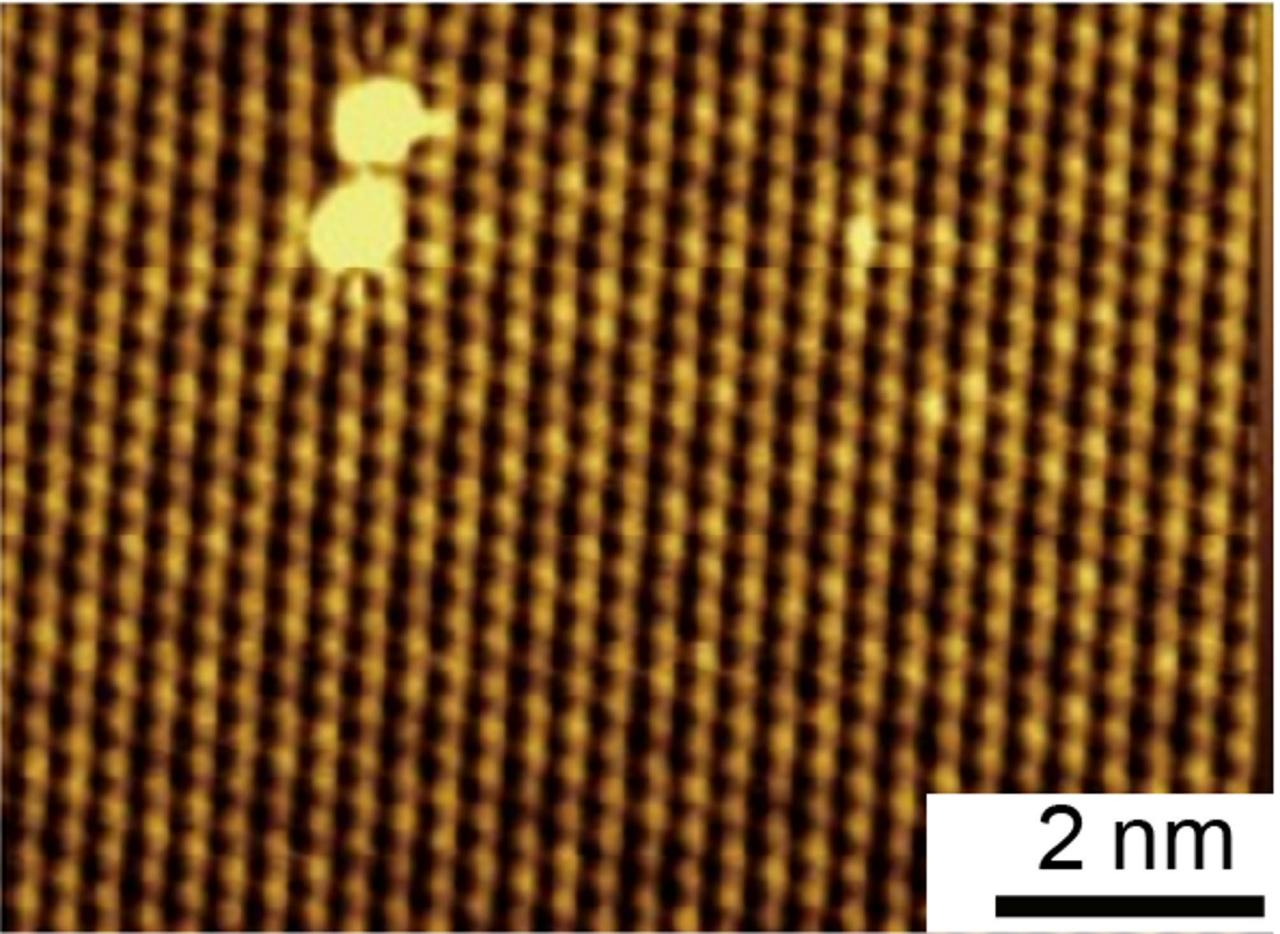
STM image of the cleaved surface of NbSe_2_, showing a 1 H phase with a 3 × 3 reconstructed structure (V_s_ = − 100 mV, I_t_ = 60 pA, and scale bar length = 2 nm).

A NbSe_2_ surface was obtained by cleaving the crystal sample in a vacuum. The STM image of the 2 H phase with a superstructure corresponding to a 3 × 3 reconstruction is shown in Fig. 1, which originates from CDW phase formation^14–16^. We identified the PAT effect based on changes in STS measurements after RF injection at the tunneling junction. Before presenting the experimental results, we outline our model for the RF effect on the STS, based on PAT theory. PAT was initially proposed to be an inelastic tunneling process involving multiple-photon adsorption and emission. Thus, replicas of the original tunneling peak appear at the positions shifted by a multiple of the photon energy^24^. The perturbation potential introduced by the RF (photon energy of ${\hslash}\omega$) is expressed as $\:{eV}_{\text{A}\text{C}}\text{cos}\left(\omega\:t\right)$. The original tunneling conductance *G*_*orig*_(V_s_) without RF changes into $\:G\left({V}_{\text{s}},{V}_{\text{A}\text{C}}\right)\:$at a tunneling bias of V_s_.

1 $$G({V}_{\text{s}},{V}_{\text{AC}})=\sum_{l=-{\infty}}^{{\infty}}{J}_{l}^{2}(\frac{e{V}_{\text{AC}}}{{\hslash}\omega}){G}_{orig} ({V}_{\text{s}}-\frac{l{\hslash}\omega}{e})$$

where *J*_*l*_ is the Bessel function of the first kind^27^. In the original report, it was understood that inelastic tunneling peaks involving the absorption and emission of RF photons appear on both sides of the initial peak with an energy shift of ± n ${\hslash\:}\omega$ where n is the number of photons involved in the inelastic process.

While the photon energy level determines the spacing between multiple peaks in PAT, the intensity of the peaks is controlled by V_AC_ contained in the term *J*_*l*_^2^(*e*V_AC_/ℏω) of Eq. (1). It exhibits oscillation with V_AC_, whose frequency and amplitude change with *l*.

Kot and coworkers demonstrated the PAT process for the vanadium surface at a cryogenic temperature using an STM setup and a superconducting material. They injected RF whose frequency is higher than 65 GHz^26^. The QP peaks split as the V_AC_ increases. The number of replica peaks and their intensity profile were well reproduced using the Tien-Gordon theory. Individual replicas shifted from the original peaks by ℏω are demonstrated in STS.

**Fig. 2:**
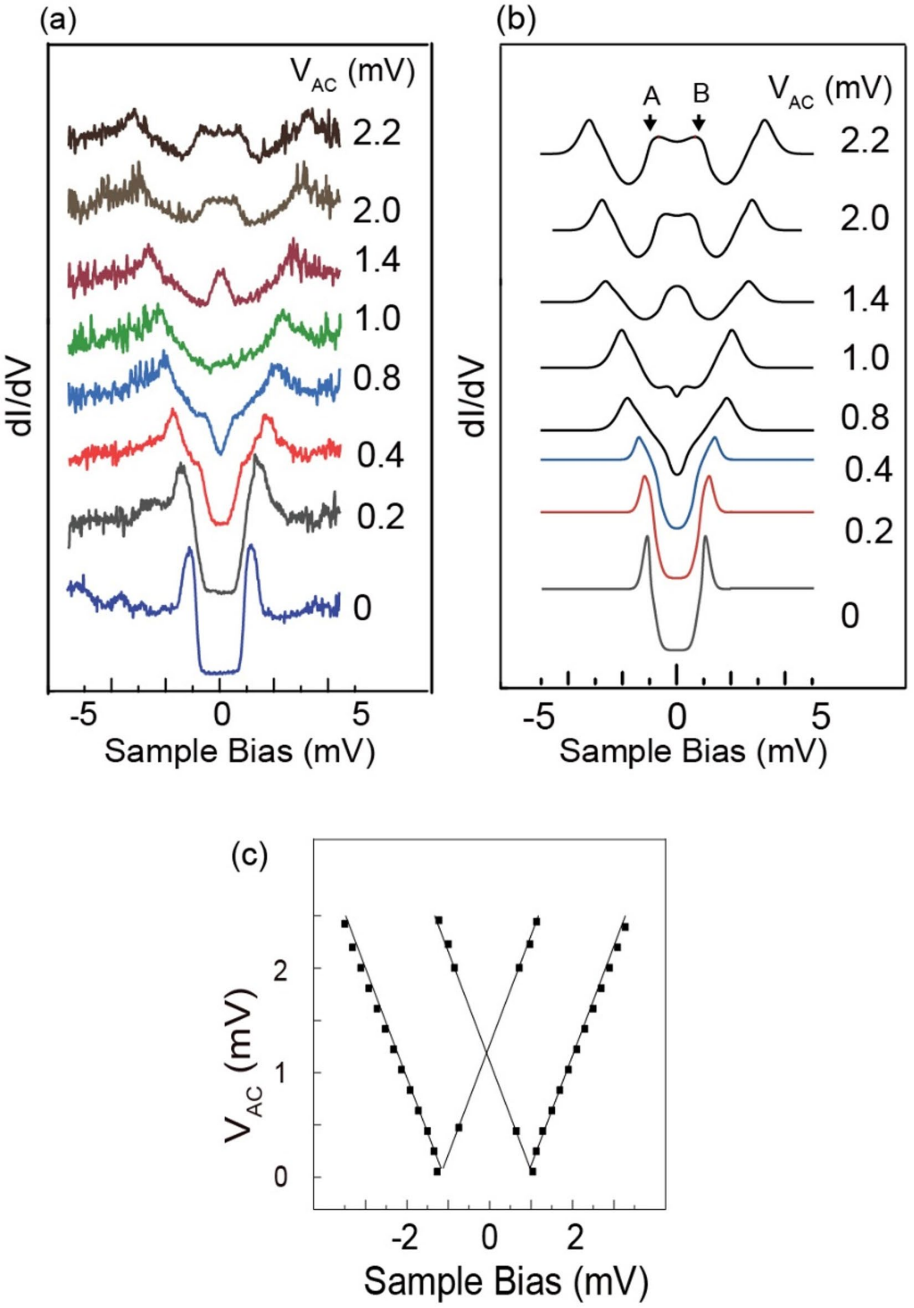
(**a**) Experimental results of the dI/dV for the NbSe_2_ surface (2 H CDW phase) with the power of RF (1 GHz) at the tunneling junction (indicated as V_AC_ on the right side of each plot. (**b**) Simulated dI/dV based on Eq. (1), whose eV_AC_ parameters are indicated as V_AC_ for each plot. (**c**) Variation of the energy position of the dominant peaks of (**b**). For each V_AC_, the energy positions of the majority peak are indicated by a small square. The linear line is the fitting result of the least-square method.

Our experimental results for the 2 H phase of the NbSe_2_ superconductor surface with the 3 × 3 superstructure are shown in Fig. 2(a), which displays a series of STS plots with varying V_AC_ of the RF (1 GHz). The starting STS without the RF injection is shown at the bottom of Fig. 2(a), whose quasi-particle (QP) appears at ± 1.0 mV. We can model this spectrum using a Gaussian peak and a step function, which were previously employed^28^. The results are shown at the bottom of Fig. 2(b); the step function staircases at ± 1.0 mV and the width of the Gaussian function is 1.0 mV.

The width of the STS peak observed on the metal surface is determined by the system temperature, the modulation voltage for the lock-in amp detection, and the intrinsic peak width^29^. The following equation gives the peak width of W.

2 $$W=\sqrt{{\left(5.4\frac{{k}_{B}T}{e}\right)}^{2}+{\left(1.7{V}_{mod}\right)}^{2}}+{W}_{int}$$

where *k*_*B*_ is the Boltzmann constant, T is the system temperature, V_mod_ is the modulation voltage (rms), and W_int_ is the intrinsic width of the peak. The QP peak broadening can be attributed to the combined effects of these three factors. The thermal broadening originates from the fact that the STM tunneling current is in a closed circuit and thermal equilibrium with the system. We need to lower the system temperature to obtain a narrower peak width, which is determined by the capability of the cryogenic cooling system. A more detailed discussion of the peak width was made by Frenke et al.^28^.

The RF electric field at the tunneling junction, V_AC_, was calibrated and correlated with the output at the RF generator (= V_AC_gen_) using the surface state of Ag surface, which was previously demonstrated^20,21^, and the details of our analysis are shown in Supplemental Information (SI). This process can obtain the RF signal’s power- and frequency-dependent transmission functions and the V_AC_ at the tunneling junction.

With the increase of V_AC_, each QP peak splits into two, one of which approaches the Fermi level, and the other separates from it. Among the four components that appeared after the RF injection, two components separating from the Fermi level are the dominant peaks for all V_AC_. The energy shift from the original peak is linear with V_AC_. The other two components approaching the Fermi level meet at V_AC_=1.0 mV, and it becomes an enhanced peak at V_AC_=1.4 mV near the Fermi level. For V_AC_ above 1.4 mV, they move toward the opposite side of the original peak position.

We simulate the evolution of the QPs of the NbSe_2_ with V_AC_ using the PAT model and Eq. (1). We assumed that the variable *G*_orig_ in Eq. (1) is modeled with Gaussian peaks and step function as stated above. Gaussian and step functions were broadened to fit the experimental spectrum. V_qp_ was set to 1.0 mV. The reason of the peak broadening of the STS spectra was explained above. The calculation was done with various V_AC_, including the ones used in Fig. 2(a). The simulated results, as shown in Fig. 2(b), reproduce the experimental results well, both in terms of energy position and peak shape. The components fanning out from two QP peaks (separating from the Fermi level) are clearly observed as an enhanced feature, while the components initially approaching the Fermi level are enhanced after crossing the Fermi level. The peaks at the top plot marked A and B are originated from positive and negative QP peaks, respectively.

The STS peak positions obtained at different V_AC_ in the simulation results are summarized in Fig. 2(c). We calculated the *J*^2^ value for V_AC_ from 0 mV to 2.4 mV with 0.2 mV increments. Solid squares indicate the positions of the peaks identified in the simulated STS. Due to the broad peak width, some features near the Fermi level are not well resolved and omitted. We fit the results with the least-square fitting method, whose result is shown by straight lines in the figure. The slope, Δ(position in STS)/Δ(V_AC_) is 1.0 ± 0.01.

Though experimental results show clear peaks which the PAT model reproduces well, the spectra variation with the RF injection is significantly different from the result of Kot and coworkers introduced above^26^, in which each QP peak separates into multiple peaks, which are separated by the photon energy. The individual peak corresponds to the number of photons involved. They correspond to $\text{n}{\hslash\:}\omega\:$, which are the photons included in the tunneling process.

**Fig. 3:**
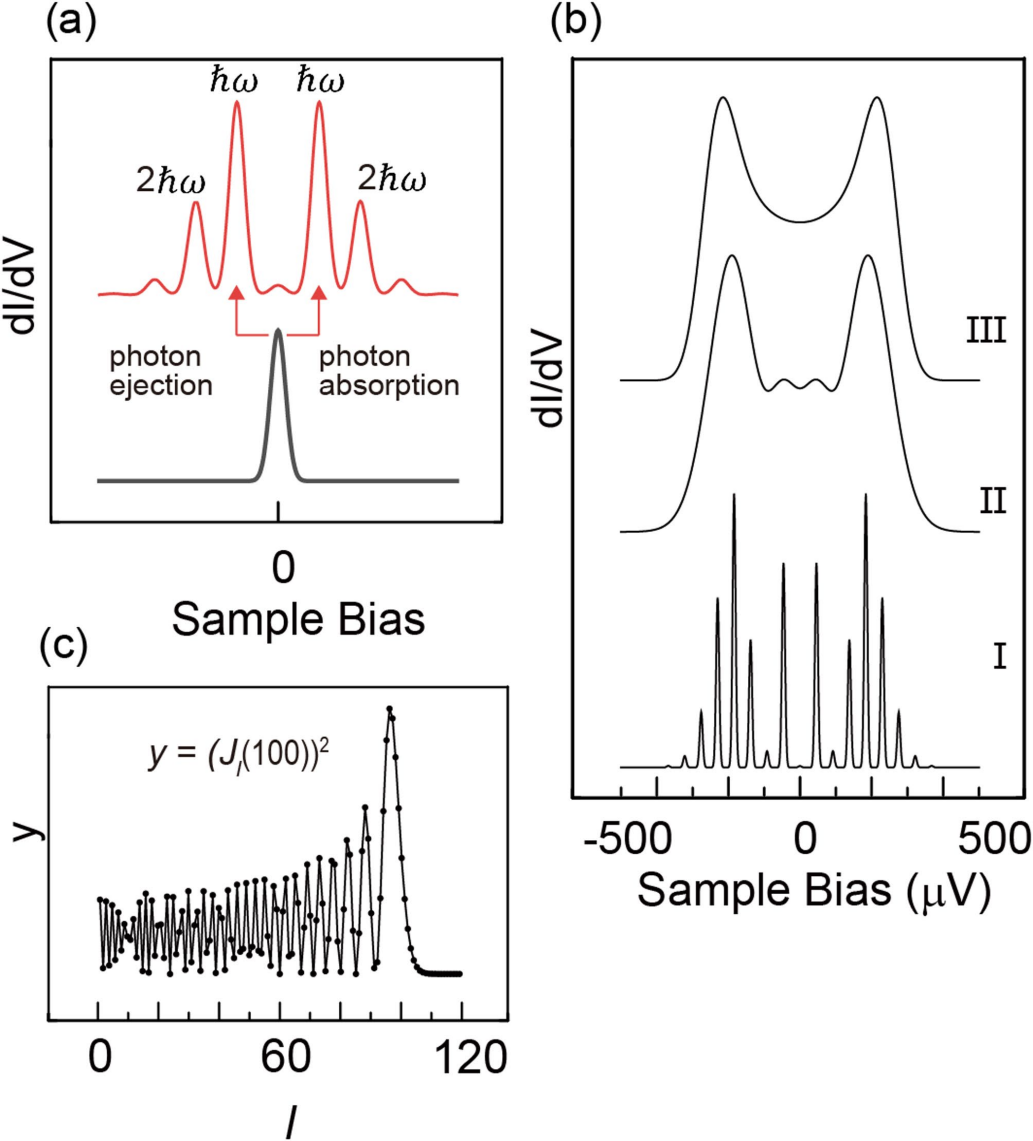
(**a**) Schematics of a PAT process involving absorption/emission of multiple photons (photon energy = ${\hslash\:}\omega\:$), whose original peak is plotted by a black line at the bottom as a Gaussian peak. (**b**) PAT simulation based on Eq. (1). Original Gaussian peak (peak position at the Fermi level) with FWHM width of 20 µV (I) and 100 µV (II, III) are changed with the RF of ${\hslash\:}\omega\:$=41.3 µV (I, II) and 4.13 µV (III), whose eV_AC_ is 250 µV (I, II, III). (**c**) A plot of the square of the Bessel function (*J*_*l*_(eV_AC_/ℏω))^2^ vs. *l* assuming eV_AC_/ℏω = 100.

To understand the mechanism of the discrepancy, we present several simulation examples in Fig. 3 based on the PAT model and Eq. (1); a more elaborate simulation can be found elsewhere^26^^,30–32^ The original Tien-Gordon model is sketched in Fig. 3(a) for a case involving only a few photons. When the number of photons increases, the simulated peak shape becomes much more complex, as shown at the bottom of Fig. 3(b). We assume a starting Gaussian peak at the Fermi level and a full width at half maximum (FWHM) of 20 µV. The RF photon energy ${\hslash\:}\omega\:$ is 41.3 µV (10 GHz) and eV_AC_ is 250 µV. RF signal injection forms multiple peaks with a spacing ${\hslash\:}\omega\:$ = 41.3 µV. The positions are shifted from the elastic peak by $\pm\:n{\hslash\:}\omega\:$, and their corresponding energies are determined by the photon energy, independent of V_Ac_.

When the starting Gaussian peak is broader (D = 100 µV), most of the sharp peaks are invisible, and only the prominent peaks at ± 250 µV and the other peaks at ± 50 µV are visible (see plot II of Fig. 3(b)). We further change the parameters, and the photon energy is changed to a lower energy (${\hslash\:}\omega\:$ = 4.13 µV corresponding to 1 GHz RF). The peaks near the Fermi level further decreased in height from Plot II, as illustrated in Plot III of Fig. 3(b).

This decrease in the number of sharp peaks from I to II (also from II to III) is mainly due to the shrinking energy spacing between the different *n* components. When the spacing is small, only the envelope of multiple peaks of the PAT process can be observed. Thus, the final spectrum would be much smoother than Plot I of Fig. 3(b). A similar effect can be expected both for the widening of the original peak and the lowering of the RF photon energy. This smooth feature cannot be attributed to the broadening of the tunneling spectrum because no spectrum broadening mechanism has been incorporated into Eq. (1).

We consider why the two peaks at ± V_AC_ are enhanced in Fig. 2. The intensity of the sharp peak in Plot I of Fig. 2(b) shows an oscillation with V_AC_, whose periodicity and maximum intensity changes with *l*. However, when *l* becomes large, like one hundred, the change with neighboring *l* becomes negligible. The smooth spectrum of Plot III of Fig. 3(b) is derived from this effect involving several hundreds of photons. The intensity of each inelastic component can be estimated using the Bessel function. We examine (*J*_*l*_(eV_AC_/ℏω))^2^ vs. *l* plot where the former term is included in Eq. (1). An example of (*J*_*l*_(eV_AC_/ℏω))^2^ vs. *l* for eV_AC_/ℏω = 100 is plotted in Fig. 3(c). The (*J*_*l*_(V_AC_/ℏω))^2^ oscillates with *l*, but the envelope increases slowly and attains its maximum value at *l* = 96, which is close to V_AC_/ℏω = 100. It thereafter rapidly decayed as *l* continued to increase. The rapid decrease in (*J*_*l*_(V_AC_/ℏω))^2^ for *l* > *e*V_AC_/ℏω is due to the negligible excitation probability for the case where the excitation energy exceeds eV_AC_, which is the case for the V_s_ > ± eV_AC_. The appearance of the maximum value near |V_s_| = ~V_AC_ is why we observe an enhanced peak. Thus, it can fit well with a linear function with a slope of ~ 1 for the QP energy shift vs. eV_AC_ indicated in Fig. 2(c).

**Fig. 4:**
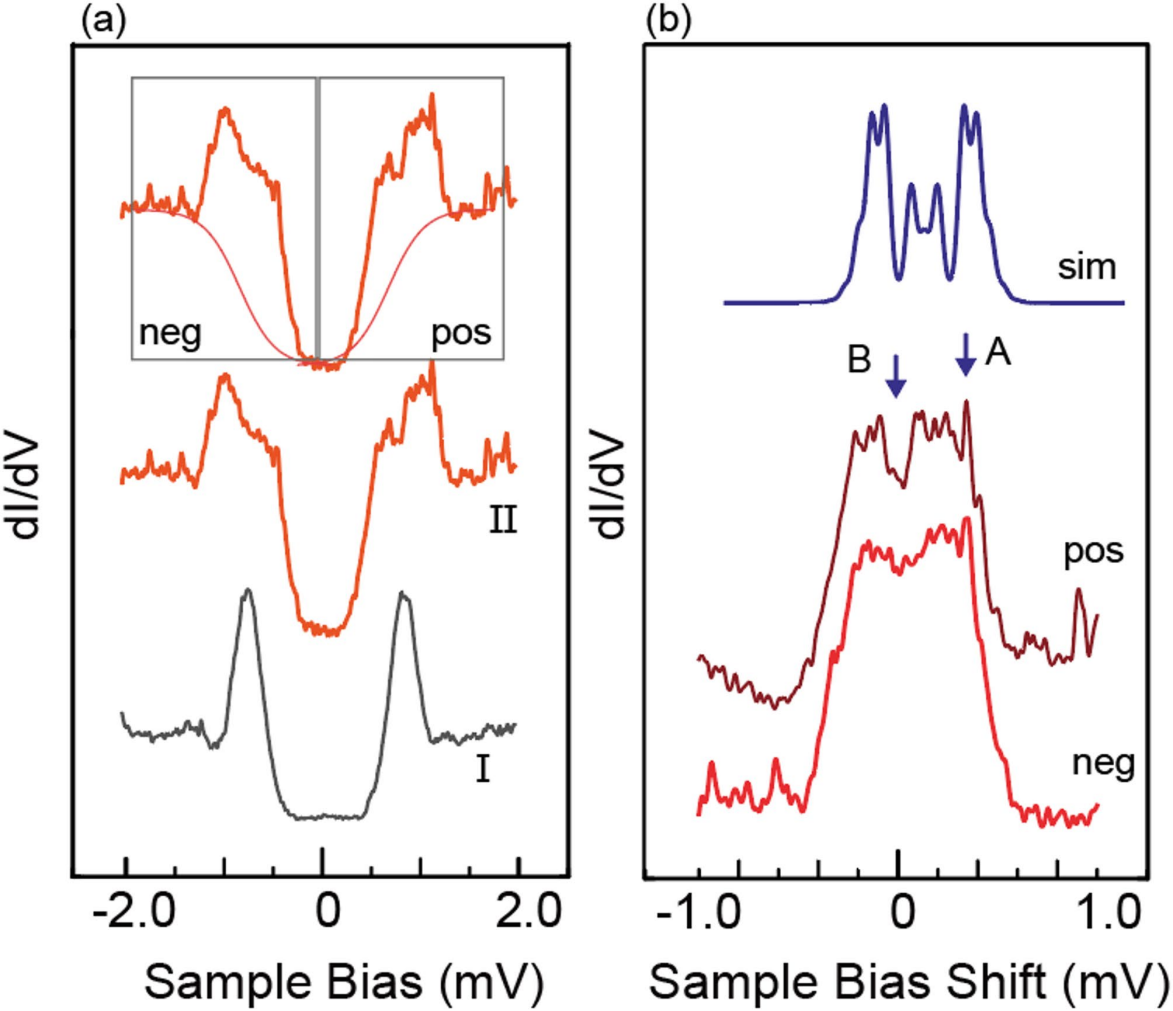
(**a**) STS spectrum of the NbSe_2_ 2 H phase surface, I, and after injecting RF (15 GHz, V_AC_ is 250 µV), II. We specify the background and two II components analyzed in (**b**) on the top panel. (**b**) The magnified plot of the top panel of (**a**). The x-axis represents the shift from the original QP position without RF. The top panel shows a simulation result obtained with the PAT method of Eq. (1). (parameters are in the main text).

To show the photon energy effect in our experiment, we show the case when higher frequency RF (${\hslash\:}\omega\:=62.0\:$µV, 15 GHz) is used. Experimental results are shown in Fig. 4. The QP feature in STS is shown as Plot I of Fig. 4(a), which changes into Plot II with the RF possessing the electric field at the junction V_AC_ =250 µV. The split peak shows a more complex feature than Fig. 2 obtained with 1 GHz RF. We subtracted the step function and compared the positive and negative QP for more detail. The background and the negative/positive area are specified in the top plot of Fig. 4(a). In the magnified spectra of negative/positive peaks in Fig. 4(b), after the background subtraction. The small features for positive and negative regions in Fig. 4(b) are reproduced with the simulation result at the panel’s top. It is intriguing to notice that the features marked by A and B appear similarly in both plots. These features do not mirror the Fermi level but shift equivalent energy from the original peak positions. To show that the minor features are free from noise, we check the sharp peak at position A and a dip at position B, as indicated in Fig. 4(b), which are clearly visible in the negative/positive spectrum. If they are the features of the superconductor, they should appear in the mirror position around the Fermi level, not a parallel shift. Thus, the inelastic feature corresponding to the multiple photon process is used for the case with RF of 15 GHz. This result confirms that the smearing of the peaks to a smooth spectrum is due to the shrinking spacing between multiple components.

**Fig. 5:**
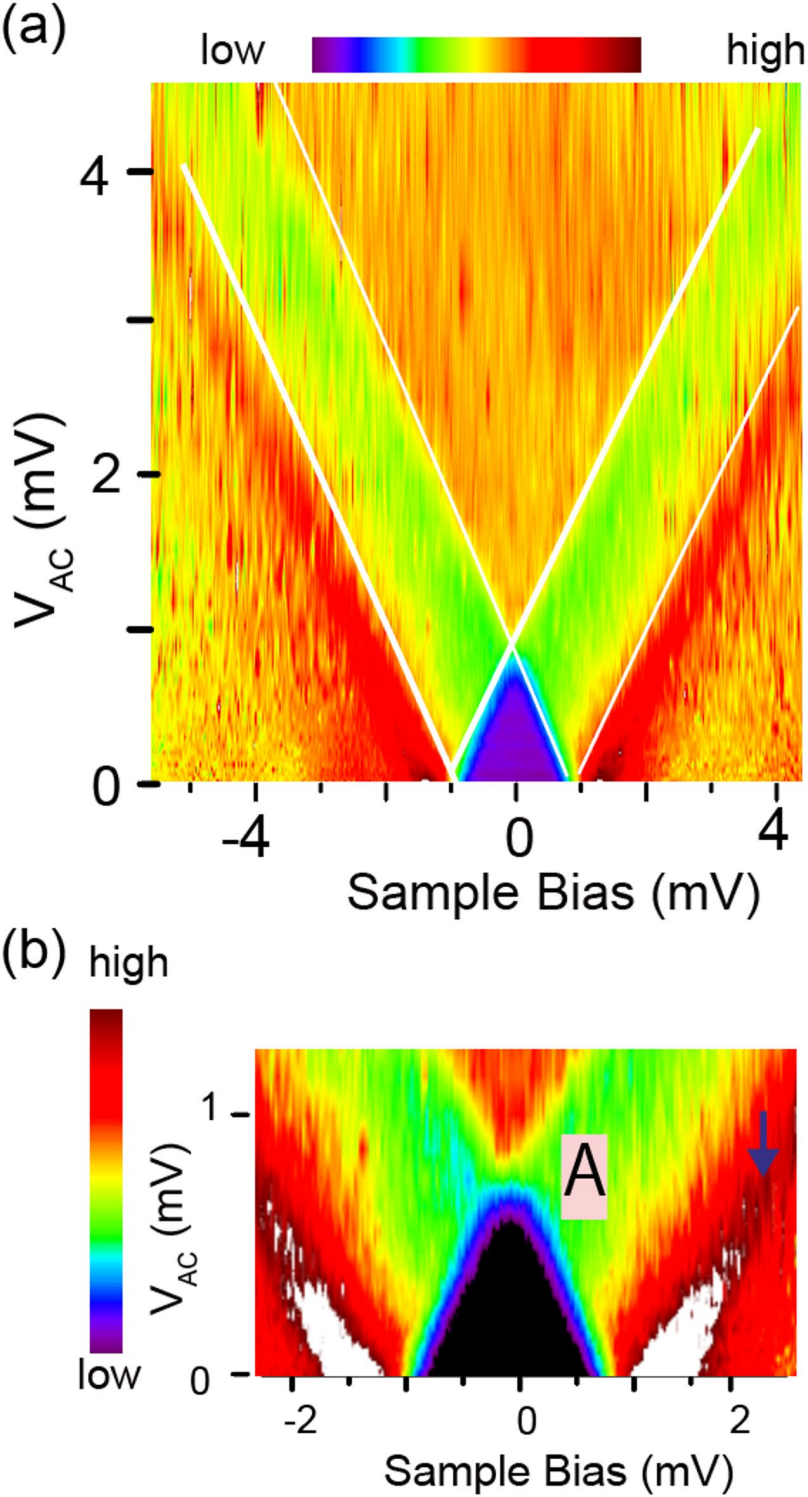
(**a**) Color mapping of the STS conductance shown in Fig. 2(a). The x-axis represents the sample bias, while the y-axis represents the RF electric field at the output. The color code adopted is shown at the top. (**b**) Magnified map of the small RF power area in (**a**). Mark A shows the position of the crossing of two inward components.

Using color mapping, we can observe the variation in the STS results with the RF power (Fig. 5(a)), which is converted from Fig. 2(a) obtained with the 1 GHz RF. The x-axis in Fig. 5(a) represents the sample bias voltage, while the y-axis represents the V_AC_. The dI/dV value is colored based on the color code at the top of Fig. 5(a). The color of the outward branch shown in Fig. 5(a) changes from red to yellow. The superimposed straight white line, which serves as an eye guide, confirms that the split energy is proportional to the RF electric field.

The inward branch approaching the Fermi level shows an intriguing intensity behavior before and after crossing the Fermi level. This can be visible in the magnified image of Fig. 5(b), in which the crossing occurs near mark A. After crossing, the peak intensity suddenly increases, which is visible as a color change from violet to yellow. Such an increase in intensity cannot be reproduced by Eq. (1). It might be caused by an interference of the two components, but we have to examine it further.

We then examined how the chemical environmental changes affect the RF-induced STS results. For that purpose, we created the 1 T phase area from the 2 H phase of the NbSe_2_ surface; the 1 T phase area exhibited a characteristic superstructure of √13 × √13 R13.9^o^, while the 2 H surface exhibited a 3 × 3 superstructure. The superstructures originated from the CDW^33–35^, indicating the presence of strong electron interactions^36,37^.

**Fig. 6:**
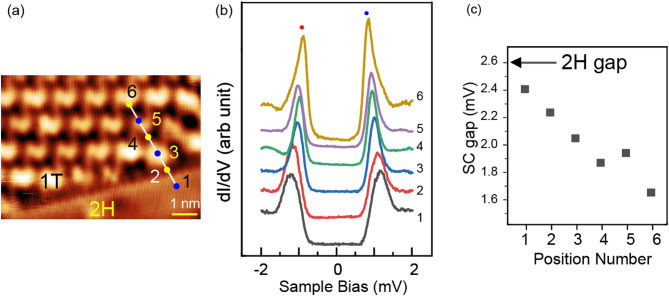
(**a**) STM topographic image of the NbSe_2_ surface at the domain boundary of the 2 H 3 × 3 and 1 T √13 × √13 superstructures. (V_s_ = − 100 mV, I_t_ = 50 pA). (**b**) Position-dependent variation of the STS results without RF injection. (**c**) The gap energy at each position at marks 1 − 6 specified in (**a**).

We formed the 1 T phase by applying a pulse with an amplitude of 3 V and a duration of 10 ms, as reported by Bischoff and coworkers^33^. The coexisting newly created 1 T phase and the original 2 H phase are illustrated in Fig. 6(a), where the √13 × √13 R13.9^o^ reconstruction is visible in the upper part of the image (the lower part corresponds to the 3 × 3 2 H phase, which is less visible than the IT phase with an unoptimized tip resolution). The two phases have almost the same height in the topographic image. The 1 T phase refers only to the top layer, and the 2 H phase is below the 1 T phase. As mentioned, the 1 T phase acts as an insulating layer, enabling electrons to tunnel from the 1 T top layer into the 2 H region below.

We compared the RF-induced QP separations observed in the 2 H and 1 T domains. We first measured STS data without an injected RF signal along the white line in Fig. 6(a). The measurement points were numbered (Fig. 6(a)), corresponding to the spectrum with the same number in Fig. 6(b). We determined the peak position by fitting the data with a model stated before: a step function for the SC gap and the Gaussian function^28^.

The STS series started from the 2 H phase, crossed the 2 H-1T interface, and entered the 1 T phase. The separation of the QP positions was reduced when moving from Spectrum 1 to Spectrum 6. We summarized the position-dependent SC gap (estimated from the energy separations of the two QPs) in Fig. 6(c). The SC gap decreased when the tip position shifted from the 2 H to the 1 T domain, with values of 2.4 meV for the 2 H domain and 1.7 meV for the 1 T domain. The gap size of the 1 T domain was reported to be smaller than that of the 2 H domain, first pointed out by Komori et al. using a bulk sample^34^. Liu et al. confirmed that an atomically thin 1 T film grown on the graphene surface has a smaller SC gap width than the pristine 2 H phase^38^. In addition, recent work on TaS_2_, a TMD family material, revealed an evolution of the spectra on approaching the step edge at the interface of the 1 T phase and 1 H region, where the QP separation monitors the SC gap. The SC gap shrinks when approaching the 1 T region, which is attributed to the edge mode related to the topological superconducting state^39^. These observations demonstrate that even a 1 T layer possesses an insulator nature; the spectrum of the 1 T phase is not the spilled state of the underlying 2 H phase but the one characteristic of the 1 T phase.

An alternative mechanism can explain the shrink of the superconductivity gap observed in the 1 T region from the 2 H region. Since it is unclear whether the 1T-phase NbSe_2_ preserves the double-gap superconductivity, it can change into a single-gap superconductor in the 1 T phase. We expect that the width of the QP peak will reduce, and the apparent gap width may shrink depending on which band disappears. When we fit the QP peaks in Fig. 6(b) using the step function and the Gaussian peak, the width is reduced from 0.47 mV for Spectrum 1 to 0.31 mV for Spectrum 6. This does not contradict the story we consider above. However, we have to do further investigation to conclude this model.

**Fig. 7:**
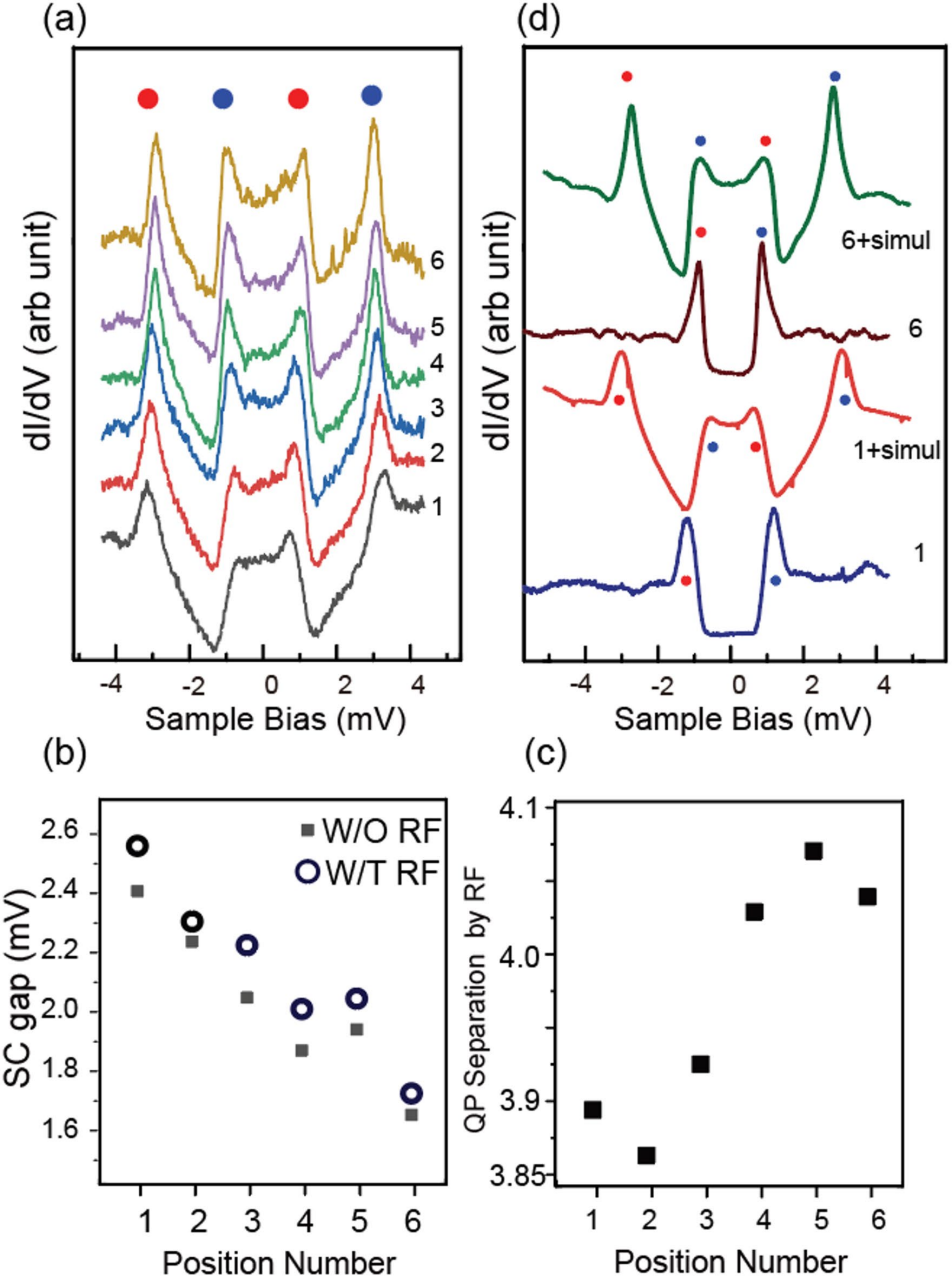
(**a**) Site-dependent variation of the QP peaks split by RF, whose V_AC_ is 19.5 mV. Positions from 1 to 6 are marked in Fig. 6(a). The circles with identical colors indicate the pair of divided peaks. (**b**) Site-dependent SC gap energy estimated by QP peaks without RF (solid circles), and the energy gap of the middle points of the separated peaks (red and blue circles) with RF (open circles). The x-axis indicates the positions are the same as (**a**). (**c**) Average of the two split energies (energy between two red dots and energy between two blue dots). Positions 1–6 are identical to (**a**) and (**b**). (**d**) Simulated spectra, starting from the Spectrum 1 and 6 of Fig. 6(b). The color circles guide the identification of the split peak, the same as in (**a**). The spectra marked 1 + simul, and 6 + simul are simulated results of Eq. (1), in which starting G is regarded as spectrum 1 and 6.

We then examined the RF-induced QP split. We provide an example in Fig. 7(a), starting on the 2 H domain with the RF condition of V_AC_ =1.95 meV and a frequency of 1 GHz. We measured RF-injected STM on the six points in Fig. 6(a). The red and blue dots shown in Fig. 7(b) split into two dots of the same color in Fig. 7(a).

In the above simulation, the positions of the split peaks were symmetric relative to the original peak position. Thus, we examined the center energy of the split peaks and compared the original peak positions without RF injection. We summarized the center energy of the split, represented by red dots in Fig. 7(b), on which the results of Fig. 7(c) without RF have been superimposed. The two series with and without the RF injection agree well, suggesting that the RF splits the original peak with equal energy separation and approaches the Fermi level.

We then analyzed the energy separation of the spectral peaks shown in Fig. 7(a), summarized in Fig. 7(c). The energy separation gradually increased from Position 1 in the 2 H domain to Position 6 in the 1 T domain after crossing the domain boundary. The density-of-state of the superconducting state increased the energy resolution, which is much better than that obtained for the thermal broadening of the Fermi edge of a typical metal^28^. Thus, while the thermal broadening of the peak width at 0.4 K is 3.5×*k*_B_T = 121 µV, an energy shift of a few tens of µV, as shown in Fig. 7(c), can be detected. We must note that the energy position at a particular surface site is determined by the sum of the site-dependent SC gap change and the RF-induced split energy change. The SC gap shrinks at the inner part of the 1 T domain, increasing the split energy. Thus, for example, the blue dots at the sample bias of 3.0 mV seem to stay at a constant energy, but the energy position and the split energies both change with the positions, and we can distinguish them clearly.

The position-dependent RF-induced peak-split energy shown in Fig. 7(c) might indicate this observation technique’s applicability to distinguish the surface’s chemical variation through different interactions between the surface and the RF signal. However, as we stated before, the STS spectra obtained on the 2 H and 1 T phase regions are different since the 1 T layer is insulating, while the 2 H layer is not, causing a double tunneling layer structure. Thus, there is a possibility that the change in the peak separation with RF injection is caused by the difference in the starting dI/dV in the two regions. To examine the effect of the shape of the original spectra on the RF-induced split energy, we compare the simulation results for the 1 T and 2 H phases. For the original spectrum G_0_ of Eq. (1), we use the experimental spectra at marks 1 and 6 of Fig. 6(c) for 2 H and 1 T phases, respectively. We calculated the STS with Eq. (1) using eV_AC_ = ± 2.0 mV to simulate the RF-induced variation. The pairs of the starting and the simulated spectrum are shown in Fig. 7(d). In the same manner as Fig. 7(a), we mark the separated peaks with the same color circles. Even though the initial peak shape and the QP energy are different for the 2 H and 1 T phases, the separations for the red circles are almost identical: 3.6710 meV and 3.6665 meV, respectively. The energy difference of 4.5 µV is orders of magnitude smaller than that shown in Fig. 7(c). We judge the effect of the spectrum shape difference in the RF-induced peak-split energy is negligible.

**Fig. 8:**
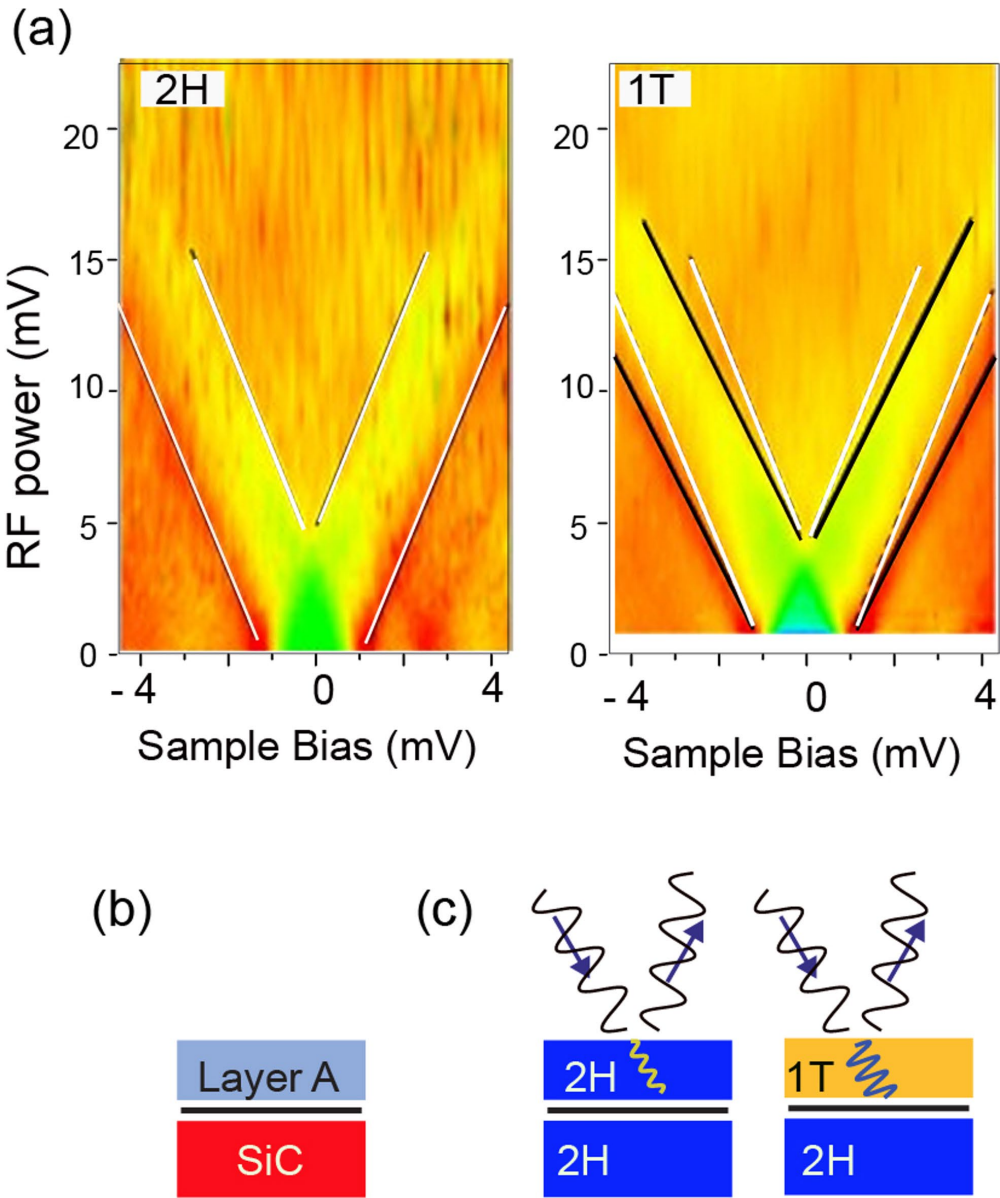
(**a**) Color maps of the STS spectra obtained by changing the RF power; the vertical axis represents the electric field at the power source. The tip is positioned at 2 H and 1 T whose spectrum is specified in the figure. Each QP peak at the bottom of the plot is split, and two enhanced peaks appear. The white line shows the linear fitting for the 2 H phase, while the black lines are for the 1 T phase. The results of the 2 H phase is superimposed onto the 1 T panale for a comparison. (**b**) Schematic of double layer configuration; various overlayer A is on SiC substrate. (**c**) Side view of NbSe_2_ schematics of 2 H and 1 T domains. The sine wave represents the incoming and outgoing RF, illustrating the difference in the penetrating signal in the top layer.

We show an RF power dependence of Fig. 7(a) feature as a color map in Fig. 8(a). We show the two positions in the 2 H and 1 T domains, in which the labels of 2 H and 1 T in the panels specify the measurement positions. In this mapping, we expressed the RF electric field at the power source (instead of at the junction) in the y-axis. The slope of the enhanced peaks is obtained by the least-square method, whose result is shown by white lines for the 2 H behavior and black lines for the 1 T behavior. We superimposed the result of the 2 H phase by white lines onto the 1 T panel. It is visible that the slope (V_AC__gen/V_samp_) for the 1 T phase is smaller than that for the 2 H phase by ∼10%, which shows a similar value estimated in Fig. 7(c).

We mention the effect of the tip condition on reproducibility. For the SC material measurement, the tip apex coating with the SC material and the atomic scale geometry affect the STS, especially the SC gap. This is true for our experiment. Thus, the series of spectra shown in the text were obtained by the identical tip.

To explain the different QP splitting energies with the same RF V_AC_ for the 2 H-1T domains, we consider a model in which the actual V_AC_ in the 2 H and 1 T regions differs. For that, we examine previous studies of near-field microwave microscopes (NFMM)^40–44^, in which the surface conductivity, the dielectric constant, and the capacity of the material surfaces are revealed with the RF injection. A spatial resolution smaller than micrometers, much shorter than the microwave wavelength, can be achieved. To explain the detection mechanism, the distribution of the electric field of the injected RF is discussed^42,45,46^.

Gu and coworkers simulated the electric field caused by the RF injection for a double-layer configuration depicted in Fig. 8(b)^47^. When the wide-gap semiconductor SiC is placed as the substrate, the RF electric field penetrates the bulk SiC. The electric field is localized beneath the tip but discontinued between the tunneling gap and sample interface owing to the significant impedance mismatch. Consequently, the bulk SiC’s electric field is smaller by more than one order of magnitude than the original value. When the atomically thin graphene layer is placed on the SiC (graphene as overlayer A)), the penetrated electric field is further reduced due to the metallic nature of the graphene layer despite its atomically thin thickness. The electric field penetrated SiC is diminished more than several orders of magnitude with the presence of atomically thin graphene. The electric field is reflected toward the tip before reaching the graphene layer.

We apply these simulation results to our case. We can consider a model of the 2 H domain as depicted in Fig. 8(c); the 1 T domain consists of a monolayer of 1 T on top of the 2 H The STS detects only the top layer’s property, and, as we described in the previous paragraph, 1 T and 2 H layers have their characteristic QP features. Thus, the electric field in the 1 T layer determines the split energy. If we compare the insulating 1 T layer and the metallic 2 H layer, a more significant electric field penetrating the top layer is expected when the top layer is 1 T phase. Since the 1 T phase is unstable in the bulk, there is no data on the electric conductivity of 1 T phases. We cannot quantitatively estimate the electric field change, but we expect a significant difference in the electric field penetration. The difference is the origin of the more substantial split of the QP peaks with the same RF power for the 1 T phase than the 2 H phase.

We consider that the PAT on SC works as the microscope, like NFMM, sensing the minute variation of the near-field, RF-induced electric field caused by local chemical environments.

## Conclusion

The injected RF signal (1 GHz and 15 Hz) at the tunneling junction makes the QP states at the end of the SC gap split, and the energy position of the split peak changes linearly with the increase of the RF power, V_AC_. However, the spectrum of the split peak is a smooth curve with two enhanced peaks at the shift position near eV_AC_ instead of discreet replica peaks separated by the photon energy. The behavior was well reproduced by a simulation using the PAT model, revealing that the disappearance of the sharp peaks is the smearing of the multi peaks by broadening the original peak width and/or the shrink of the spacing with lowering the photon energy. In addition, we successfully revealed the origin of the two enhanced peaks near eV_AC_, which is deduced from the Bessel function. We apply RF injection STS to investigate property changes at the boundary of the 2 H and 1 T phases. We found the superconducting gap decreases when we move the tip from the 2 H domain into the 1 T domain. Interestingly, the linear energy shift of the RF-induced peaks with RF signal’s power shows different coefficients between the 2 H and 1 T phases. We concluded that this change is deduced from a difference in the actual VAc on the two domains, originating from different dielectric constants originating from the insulating nature of the 1 T layer, resulting in a different shielding efficiency for the electric field of RF.

## Methods

**Fig. 9:**
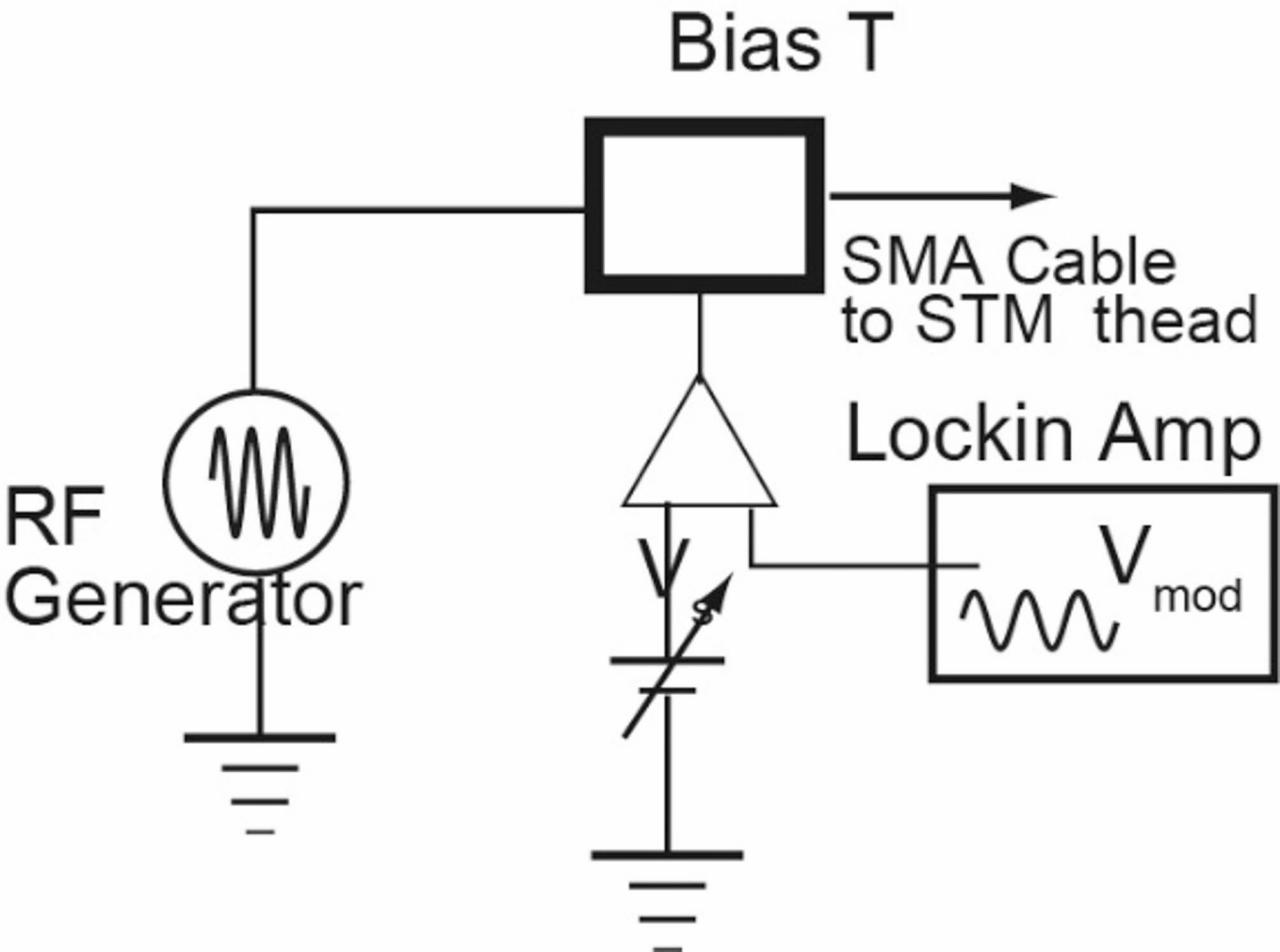
Schematic showing the injection of the RF signal at the tunneling junction. Tunneling bias is the sum of DC voltage, modulation voltage detected by the lock-in detector, and RF voltage.

Our experiments employed an ultrahigh vacuum STM (Unisoku, Japan) in which the sample was cooled to 400 mK using ^3^He. The sample temperature was maintained at 400 mK throughout the experiment. We used PtIr wire as the material for the STM tip. The schematic of the RF injection at the STM tunneling junction is depicted in Fig. 9. We introduce the RF field through SubMiniature version A (SMA) cable, which starts from the output of the RF generator and ends at the anchor point in the UHV using high-frequency compatible connectors, including the feedthrough between the ambient and the vacuum. The anchor point in the UHV is located a few inches from the STM head. From the anchor point, the cable was switched to a thin wire to prevent the mechanical vibration caused by the solid SMA cable. Because the length of this section is short, no significant RF signal loss occurs along it.

The RF signal (1–15 GHz) is introduced to the tunneling junction by superimposing the signal from the RF generator onto the DC tunneling bias voltage using a bias tee connector. The effect of RF injection on the dI/dV spectrum of the QP peaks shown in this manuscript is visible in the continuous wave mode (without RF on/off chopping and lock-in-amplifier). Thus, we continued the RF signal injection during the dI/dV measurement. We superimposed a low-frequency (∼300 Hz) sinusoidal modulation voltage on the bias voltage to enable STS measurements. The tunneling bias voltage was applied to the STM tip, and the tunneling current was provided from the sample.

## Electronic supplementary material

Below is the link to the electronic supplementary material.

Supplementary Material 1

## Data Availability

All data are available in the main text. Any additional data are available from the corresponding author upon request.
