# Supplementary material for: RF injection scanning tunneling spectroscopy of a superconducting NbSe2 surface
Source: Sci Rep. 2025 Jul 1;15:20622. doi: 10.1038/s41598-025-07203-2 (PMC12216885; doi:10.1038/s41598-025-07203-2)
Supplement: Supplementary file 1 — Supplementary Material 1 [file 41598_2025_7203_MOESM1_ESM.pdf]

Supplementary Information.

RF Injection Scanning Tunneling Spectroscopy of a Superconducting $NbSe_2$ Surface

Md. Arafat Ali [1], Zhipeng Wang[1,3], Mohammad Ikram Hossain[1,3], FerdousAra[2],

Syed Mohammad Fakruddin Shahed[2], and Tadahiro Komeda ,[2,3]*

[1)]Department of Chemistry, Graduate School of Science, Tohoku University, 6-3, Aramaki Aza-Aoba, Aoba-ku, Sendai 980-8578, Japan

[2)]Institute of Multidisciplinary Research for Advanced Materials (IMRAM, Tagen), Tohoku University, 2-1-1, Katahira, Aoba-Ku, Sendai 980-0877, Japan

[3)] Center for Spintronics Research Network, Tohoku University, 2-1-1 Katahira, Aoba-ku, Sendai 980-8577, JAPAN

[*]Corresponding Author: tadahiro.komeda.a1@tohoku.ac.jp

Table of contents.

S1. Determination of transmission function and electric field at the tunneling junction

S1. Determination of transmission function and electric field at the tunneling junction

Due to RF components (such as cables, connectors, and feedthrough), the transmission of the entire RF signal line has a large frequency dependency. Since this frequency-dependent transmission makes spectrum analysis difficult, we have to tune the RF generators' output so that the RF power at the tunneling junction remains constant.

To compensate for the frequency-dependent influence of the transmission, we tuned RF power at the generator by following the procedure reported previously{Paul, 2016 #6196}. First, we quantified V_{AC} utilizing the rectification of nonlinearity in the I-V characteristic obtained for the Ag(111) surface state. The Ag(111) surface was prepared with repeated Ar+ ion sputtering and annealing at 500 °C. The prepared surface shows the monolayer steps with a hexagonal atomic structure (Figure S1(a)). The dI/dV spectrum obtained on the Ag(111) surface (black spectrum in Figure S1(b) shows the step-like structure, which indicates the onset of the (two-dimensional) surface state. The application of the RF signal (output power of -5 dBm) broadens the step structure (blue spectrum). We fitted both spectra with the convolution of an arcsine function and thereby determined the V_{AC} amplitude to be about 25 mV.

Next, we acquired the RF transmission on Ag(111) surface by sweeping frequency at the constant RF generator power of +5 dBm. The sample bias was adjusted to the Ag(111) surface state onset of -70 mV (see Figure S1(b)), while applying RF signal for rectification of nonlinearity in the I-V curve. Here, we set the tunneling current to 500 pA, which is higher than that typically used in our measurement (10 pA). The higher tunneling current increases the V_{AC} and therefore allows us to obtain accurate relative intensity of the RF signal over the entire frequency range, especially in the frequency region in which the signal is strongly attenuated within the RF line. We modulated the amplitude of the RF signal in chopping scheme at frequency (f_{mod}) of 431 Hz to detect the V_{RF} - induced tunnel current with lock-in amplifier synchronized at f_{mod}. The extracted RF signal amplitude ($V_{lock\text{-}in}$), which is proportional to V_{RF}, was divided by source power to determine the RF transmission. Figure S1(c) shows a measured RF transmission on Ag(111) surface at constant P_{RF} = +5 dBm. The relation between V_{RF} and $V_{lock\text{-}in}$ is described as $V_{RF} = V_{lock\text{-}in} + C$ in any frequency[1], this constant values was obtained from STS measurement of step broadening in Ag(111) surface. By using the evaluation of $V_{lock\text{-}}$

in with sweeping RF power at constant frequency and C, we can obtain a P_{RF} corresponding to any V_{AC}.

To compensate for the frequency-dependent variation of RF transmission, we tuned the generator power as plotted in Figure S1(d), which was calculated from the obtained transmission. We applied the calibrated P_{RF} extracted from Ag(111) surface transmission to deduce V_{AC}.

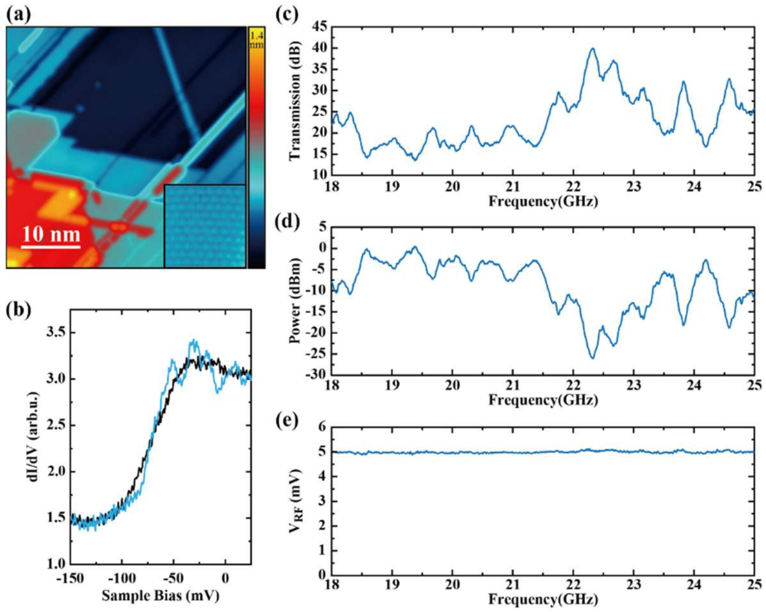

Figure S1. Compensation of RF transmission at the tunneling junction. (a) Typical large-scale STM image of Ag(111) surface (scanning condition: V_{DC} = 0.9 V, I = 18 pA). Inset shows atomic resolution image (scanning condition: Vs = 70 mV, I = 500 pA). (b) dI/dV spectra of step-like Ag(111) surface state without (black curve) and with (blue curve) RF signal (RF generator power of －5 dBm, f = 19 GHz). The applying continuous RF signal broadens the step-like surface state of Ag(111), which indicates that the V_{RF} amplitude at the tunnel junction is about 25 mV. (c) RF transmission of frequency range in 18 GHz – 25 GHz at constant RF source power of 5 dBm. (d) The RF source power for constant V_{AC} at tunnel junction. (e) The constant V_{RF} amplitude of 5 mV was achieved utilizing compensated source power.
